# Recent Developments in Identification of Genuine Odor- and Taste-Active Compounds in Foods

**DOI:** 10.3390/foods10071628

**Published:** 2021-07-14

**Authors:** Enrique Durán-Guerrero, Remedios Castro Mejías

**Affiliations:** Analytical Chemistry Department, Faculty of Sciences-IVAGRO, University of Cádiz, Agrifood Campus of International Excellence (ceiA3), Pol. Río San Pedro, Puerto Real, 11510 Cadiz, Spain; enrique.duranguerrero@uca.es

Both aroma and taste are crucial quality criteria for food products, having a great influence on our consumption behaviour. As a consequence of this, nowadays, the accurate quantification and identification of the compounds related to flavour in the food industry is a growing need. In recent years, the development of highly sensitive and selective analytical methods has implied a significant improvement in the identification of the characteristic odor- and taste-active compounds of particular foods. In spite of this, as the concentration of these compounds is usually really low, previous highly efficient preconcentration approaches are generally necessary.

This Special Issue is focused on the recent analytical developments for the identification of the compounds responsible for odour and taste in foods, including methodologies for isolating volatile compounds from complex food matrices and the possible relationships between flavour compounds and industrial processing or cultivar-dependent conditions. As a result, this Special Issue includes nine valuable scientific contributions, among which a study about compounds related to taste and colour in blueberry can be found. In it, Aliaño-González et al. validated and optimized a novel method based on ultrasound-assisted extraction for the quantification of total anthocyanins and total phenolic compounds in a fruit, blueberry, with an increasing consumption in these recent years thanks, in part, to its high content in bioactive compounds [1].

Regarding compounds responsible for taste and colour, in this case in wine, Schwarz et al. have carried out the study of different fractions from Amontillado sherry wine obtained using high-speed counter-current chromatography [2]. Forty-two phenolic compounds and furanic derivatives have been identified by means of High Pressure Liquid Chromatography-Diode Array Detector-Mass Spectrometry HPLC-DAD-MS, 11 of which were identified for the first time in sherry wines: 3-feruloylquinic acid, isovanillin, ethyl vanillate, furoic acid, dihydro-p-coumaric acid, 6-*O*-feruloylglucose, ethyl gallate, hydroxytyrosol, methyl protocatechuate, homoveratric acid, and veratraldehyde. 

Concerning compounds related to aroma, a methodology by high-performance liquid chromatography with diode array detection was optimized by Cebrián-Tarancón et al. to analyse intact glycosidic aroma precursors in grapes [3]. For this, briefly, 20 g of homogenized freeze-dried skin was ground to a fine powder and moisturized with 70 mL of water for 2 h at room temperature. After that, an extraction phase was carried out employing microwave. Then, a Solid Phase Extraction (SPE) was employed in order to eliminate sugars and some polyphenols, previously to the HPLC-DAD determination. Among the identified compounds, with respect to sugars, disaccharides hexose-pentose was the most abundant group, whereas monoterpendiols were the main aglycones.

As well, in the case of oenological products, Roldan at al. performed the determination by Solid Phase Extraction-Gas Chromatography-Flame Ionization Detector (SPE-GC-FID) of the volatile compounds in Palomino wines obtained under different oenological techniques (pellicular maceration with supra-extraction and use of commercial yeast strains and of glycosidase enzymes) [4]. The greatest changes in aroma and sensory profile were a result of the pellicular maceration and supra-extraction techniques. The highest content of terpenes and floral odours were found for supra-extraction techniques. This technique was the most selective technique, since it extracted terpenes and aromatic precursors but not other volatile compounds responsible for certain fatty characteristics.

From a wider perspective and using polyphenols, colour parameters, and some volatile compounds, Valcarcel et al. have carried out a study about the evolution of Brandy de Jerez aged in American oak barrels with different times of use: new, with 7 years of use (4 years containing oloroso Sherry wine and 3 years containing wine spirits) and with 32 years of use (8 years containing oloroso Sherry wine and 24 years containing wine spirits) [5]. According to the results, even after 32 years of use, the oak wood can modify the organoleptic characteristics of brandy, and no significant differences were found between aged brandies in 4+3 used casks and those aged in 8+24 used casks. From a sensory point of view, the brandies aged in used barrels were evaluated as more balanced than those aged in new casks.

With respect to other matrices different to oenological products, González-Dominguez et al. established, by means of CG-FID together with classical multivariate statistical models and artificial neural networks, the closely relationship between the inclusion of acorns in pigs´ diet and the increase in the content of monounsaturated fatty acids, mainly oleic acid, representing a first step toward the development of a suitable tool for the Iberian ham authentication and fraud detection [6].

Using a headspace sorptive extraction method coupled with gas chromatography–mass spectrometry (HSSE–GC–MS), Ruvalcaba et al. determined 37 volatile compounds in beer and compared this methodology with a previously developed and validated stir bar sorptive extraction (SBSE) method [7]. HSSE presented some advantages when it was compared to SBSE. A lower degradation of the stirring bars and sample volumes that are considerably reduced are properties of this technology. Together with this, the aroma detected by HSSE is considered as more representative of what consumers perceive when they smell a particular beer.

Headspace solid-phase micro-extraction coupled with gas chromatography was used by González-Dominguez et al. to investigate the influence of cultivar (Festival, Candonga, Camarosa cultivars) on the volatile profile of strawberries cultivated under a soilless system [8]. Festival and Candonga varieties were rich in esters, furanones, and terpenes. Among these ones, methyl butyrate, hexyl hexanoate, linalool, geraniol, and furaneol were the most abundant aromatic compounds detected in the three varieties of strawberries. According to pattern recognition chemometric techniques, geraniol and hexyl hexanoate were found to be the most significant volatiles for the discrimination of the three strawberry varieties considered in this study.

With regard to other non-chromatographic methodologies applied, in this case, indirectly to the determination of volatile compounds, Rivas et al. developed a rapid PCR method for the detection of 1,3-pentadiene non-producing debaryomyces hansenii yeast strains [9]. This is a volatile compound with unpleasant “petroleum-like” odour that is generated from sorbic acid salts added as preservatives against unwanted microbial growth when certain yeast strains are present. 3-pentadiene is usually measured by gas chromatography with mass spectrometry detection (GC-MS) or MWIR (Mid-Wave IR), but both technologies are characterized by time consumption and high costs, so this alternative represents an easier, quicker, and less expensive methodology than the previous ones.

In summary, the Special Issue “Recent Developments in Identification of Genuine Odor- and Taste-Active Compounds in Foods” shows some of the latest advances in the truthful quantification and the identification of several compounds related to aroma and taste in the food industry and how they can be influenced by the different stages and conditions under which these foods are elaborated.
